# A 3D Printing Platform for Design and Manufacturing of Multi-Functional Cementitious Construction Components and Its Validation for a Post-Tensioned Beam

**DOI:** 10.3390/ma17184653

**Published:** 2024-09-23

**Authors:** Ofer Asaf, Arnon Bentur, Oded Amir, Pavel Larianovsky, Ohad Yaacov Meyuhas, Eliad Michli, Aaron Sprecher

**Affiliations:** 1 Technion Advanced Construction Center, National Building Research Institute, Technion Technion–Israel Institute of Technology, Haifa 3200003, Israel; odedamir@technion.ac.il (O.A.); lpavel@technion.ac.il (P.L.); ohad-meyuhas@campus.technion.ac.il (O.Y.M.); eliad.michli@campus.technion.ac.il (E.M.); asprecher@technion.ac.il (A.S.); 2 Faculty of Architecture and Town Planning, Technion–Israel Institute of Technology, Haifa 3200003, Israel; 3 Faculty of Civil and Environmental Engineering, Technion–Israel Institute of Technology, Haifa 3200003, Israel

**Keywords:** 3D printing, additive manufacturing, holistic approach, post-tensioned beam, cementitious materials

## Abstract

Three-dimensional printing of cementitious materials for construction has been extensively investigated in recent years, with several demonstration projects successfully carried out. These efforts aim to leverage the printing process to achieve more efficient production of components compared to conventional concrete technologies. This includes both the process itself (eliminating the formwork stage) and the flexibility in producing complexly shaped elements. To maximize the potential of 3D printing in the construction industry, additional steps must be taken, grounded in a holistic view of the entire process. This involves integration of the production chain, including design, materials, and manufacturing of components, to create elements with optimal performance, encompassing structural, environmental, and architectural aspects. Such multi-functionality requires the viewing of 3D printing not just as a production technology but as a platform enabling the integration of all these components. To advance this approach, quantitative tools are developed to optimize the following three key components: material composition; manufacturing parameters to ensure buildability; and design tools to optimize multiple performance criteria, particularly structural and architectural shape. A demonstration component, namely a post-tensioned beam, featuring two multi-functional characteristics—structural and architectural—is designed, produced, and evaluated. The scientific concepts and research tools used to develop these quantitative design tools are multidisciplinary, including rheological characterization, control of the internal structure and composition of granular materials, simulation of the mechanical behavior of green material during printing, and the hardened properties of the components, all utilizing structural optimization to enhance performance.

## 1. Introduction

The production of construction components through 3D printing using cementitious mixes has emerged as a highly promising technology in the construction industry. Extensive research, particularly in the past decade, has led to several successful applications, albeit on a limited scale. The primary driving forces behind this technology are its potential to improve industrial productivity by saving on formworks, materials, and manpower, which is of great interest to engineering technology stakeholders, and its ability to facilitate the production of complexly shaped components, which appeals to the architectural community. These motivations have spurred significant research on the material and technological aspects of 3D printing with cementitious materials, as well as on digital fabrication and design. State-of-the-art overviews and reviews in these areas have been published [1,2,3,4,5,6,7,8].

The most intense research efforts have focused on facilitating the development of technology for 3D printing with cementitious materials, delving deeply into the properties and behaviors of material compositions that can be effectively incorporated into 3D printing systems to achieve stable layered structures, which are essential for additive manufacturing. This research has primarily addressed the fundamental physical, chemical, and mechanical characteristics of cementitious systems at a very early age—up to about 60 minutes. Notably, a rheological approach has been employed to describe and quantify the various processes occurring, adopting methodologies based on soil mechanics and soil stability [9,10,11,12,13,14,15,16,17,18,19,20,21,22,23,24,25,26,27].

Concurrently, significant efforts have been directed towards the development of digital fabrication and design methods for complexly shaped components, driven largely by architectural interests that leverage the ability of additive manufacturing technologies to produce intricate shapes [18,20,21,23,24,28,29,30,31,32,33].

A third focused research effort, largely independent of the others, has been motivated by the potential of additive manufacturing to produce efficient structural components using structural optimization (e.g., [34,35,36,37,38,39]).

Despite considerable advancements in these fields, much research has been conducted in isolation, with less attention given to a holistic approach [40]. Heywood and Nicholas recently highlighted the opportunities for a holistic approach to sustainability, analyzing activities in material science, computational design, and structural performance [41]. They concluded that while impressive advancements have been made in each area, there is insufficient interaction and bridging between them, leading to missed opportunities for optimization that could be achieved through a holistic perspective. Although Heywood and Nicholas focused on sustainability, their insights can be extended to the optimization of components for multiple performances to produce multi-functional cementitious construction components.

The objective of the present study is to explore the potential of such a holistic approach by developing and applying design tools at the levels of material, manufacturing, and performance design and integrating them to enable the creation of multi-functional components. The specific variables studied to facilitate this holistic approach— material composition, printing parameters, and design optimization—are described in detail in Section 2.

## 2. The Holistic Framework

**Figure 1:**
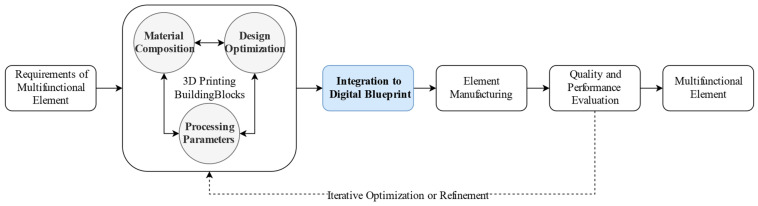
Framework for developing a holistic approach to optimize construction components produced by 3D printing with cementitious materials.

To provide a methodology for the development of design tools within a holistic approach, a comprehensive framework was established, as presented in Figure 1.

This approach considers the following three individual building blocks: material composition, design optimization, and processing parameters. While each of these blocks can be treated independently, true optimization occurs by considering the interactions between them. This perspective necessitates different optimization approaches for each building block by identifying and quantifying the links between them.

To clarify the implications of this approach, consider the optimization of mix design. The prevailing concept in many publications is to define a range of properties to set a window, often representing the upper and lower bounds of rheological properties, and determine the mix’s suitability to meet these constraints by adjusting the content and type of admixtures and other ingredients [42,43,44,45]. However, our approach adjusts the parameters of the printing process, explicitly printing velocity and the printed layers’ height/width ratio in relation to the rheological properties of the mix. This is done to successfully print an optimized design that considers the geometry’s overhanging limitations of the mix [46]. This component-specific “tailoring” approach provides a greater degree of freedom by simultaneously considering mix design and printing process interactions, enabling the use of various mix designs by adjusting the printing parameters. This method could facilitate the use of local raw materials, including recycled ones. Such optimization may also consider requirements such as the time required to print the component and the cost of materials, leveraging the degrees of freedom in the mix design.

Similar relationships can be established between the manufacturing and design building blocks to complete the array of interactions and their quantification to facilitate overall optimization.

To achieve these goals, the research plan includes the following three major components:

1. Characterization of cementitious mixes to optimize their properties for printing, resulting in quantitative parameters that are used as inputs for computational models that predict buildability;
2. Development of quantitative tools to design the printing process based on the material properties in a fresh state;
3. Development of shape optimization tools to achieve structural performance with a minimum quantity of materials.

**Figure 2:**
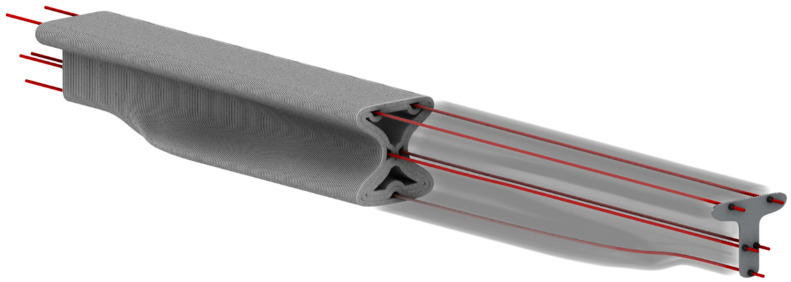
The geometry and post-tensioning scheme of the beam that was designed, fabricated, and tested to demonstrate the holistic approach.

The integration of these components into a unified digital blueprint for production was demonstrated and validated for the case of a post-tensioned beam printed as two symmetrical components with the general geometry outlined in Figure 2. The beam was designed and fabricated to verify the holistic approach. The beam was printed using the materials and tools developed in this study, and the dimensional accuracy of its manufacturing was evaluated.

## 3. Material Composition

The term often collectively used for 3D printing material in construction is concrete (e.g., [47,48,49,50,51]), which covers concretes (with aggregate sizes of more than 10 mm), as well as mortars (with maximum aggregate sizes in the range of sand—usually smaller than 2 mm). Thus, there should be a preference to use the term cementitious system or cement-based material (e.g., [24]) rather than concrete. This distinction—in particular, the differentiation between concretes and mortars for 3D printing—has several important implications. Mortars usually have higher cement content than concretes due to limitations in particle size grading, which can be more efficient in concrete because of the larger particle size [52,53].

However, adopting a holistic approach requires consideration of the limitations imposed on optimized designs, which are constrained by the need for wider cross-sections when printing with concrete. This can reduce the potential for more complex cross-section geometries, which could be more efficient in terms of structural stability and thermal resistance. Overall optimization can enable material savings in the components’ cross-sections. Thus, a balance must be struck between the overall material quantity, which could be smaller when printing with mortar, and the lower cement content when printing with concrete. This highlights the need to simultaneously optimize both the material composition and structural design of components.

### 3.1. Test Methods

Details of the experimental methods for material mix design and characterization are reported by Asaf et al. [16]. Testing of the green material included rheological characterization for 75 minutes after adding water, using an ICAR rheometer (Germann Instruments, Evanston, Illinois, USA) to determine the static and dynamic yield stresses. In parallel, the spread in the ASTM C 230 flow table (CONTROLS S.p.A., Milan, Italy) test for mortars was determined by evaluating the spread value of the mix after 25 jolts.

Additionally, early-age properties were determined by ultrasonic testing to characterize the kinetics of the processes and provide estimates of the mechanical performance by evaluating the dynamic modulus of elasticity. Ultrasonic measurements were carried out with the Test System IP-8 (UltraTest GmbH 2022, Achim, Germany), which consists of a metallic cylindrical chamber equipped with an ultrasonic measurement system on both sides that provides ultrasonic pulse velocity. The mortar mix used for printing was cast immediately after mixing into the chamber, which was kept under lab conditions identical to those of the printing process. The advantage of this setup is the ability to commence with measurement immediately after mixing to provide efficient recording within the first few minutes, which is of special significance for 3D printing.

The properties of the hardened material were determined through standard compressive and flexural tests of beams, along with special specimens produced by printing, as reported by Asaf et al. [16].

### 3.2. Materials and Mix Composition

**Figure 3:**
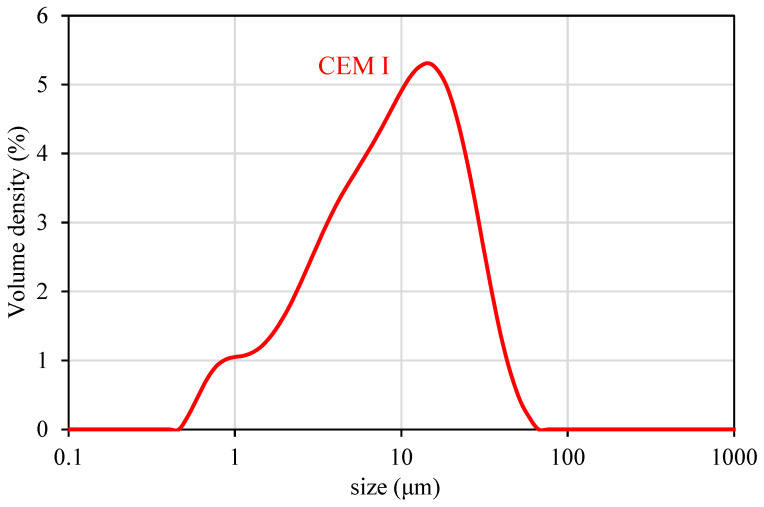
Particle size distribution of CEM I.

The printing mix was a mortar previously developed to provide adequate properties for printing, consisting of Type I Portland cement, microsilica, clay, and siliceous sand (see Table 1). CEM I Portland cement of 52.5 MPa strength grade was used. The mineralogical compositions and particle size distribution of CEM I are presented in Table 2 and Figure 3.

**Figure 4:**
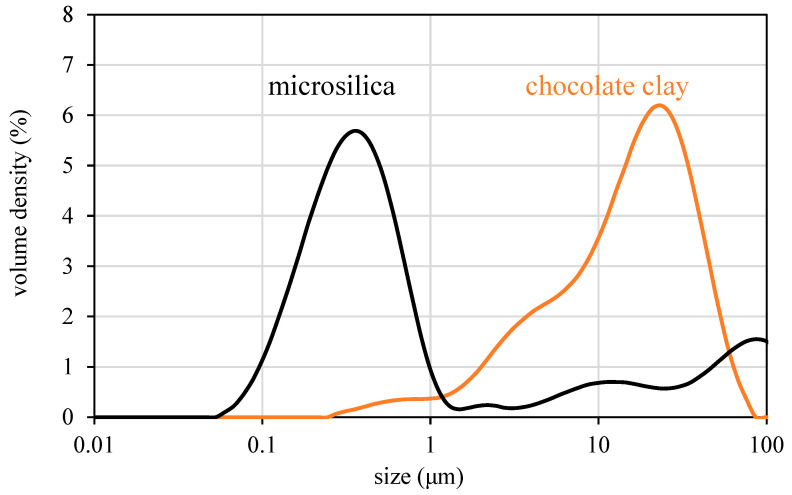
Particle size distribution of the two mineral admixtures used in this study.

The mineralogical composition and particle size distribution of the two mineral admixtures used in this study are provided in Table 3 and Table 4 and in Figure 4.

The properties of the mix were adjusted by adding low-modulus fibers to control the rheological properties and accelerating admixtures, as used in shotcrete, to accelerate the early-age reaction. Both were employed to enhance the thixotropic nature of the mix.

### 3.3. Properties of the Printing Mix

The methodology for characterizing cementitious mixes and designing the printing process is based on quantifying rheological properties, as described by Asaf et al. [16]. This methodology follows concepts set by Kruger et al. [54] and focuses on the thixotropic nature of cementitious mixes.

Rheological testing using the ICAR rotational rheometer primarily covered the behavior over the first 75 minutes, which is the period most relevant to 3D printing manufacturing. This testing was accompanied by ultrasonic testing over this period and longer—up to several days. These tests characterized mechanical parameters, yield stress values (static and dynamic), and the development of the dynamic modulus of elasticity, which served as inputs for the modeling and design of the printing process to ensure adequate flow and buildability.

To provide a basis for optimization, there was a need to resolve basic parameters of the printing mix (in particular, rheological parameters—mainly static and dynamic yield stresses) [15,16]. To study the effects of composition on these parameters, a reference mix developed in a previous study [16] was used (see Table 1), and changes in its composition and their effects on rheological parameters were studied.

**Figure 5:**
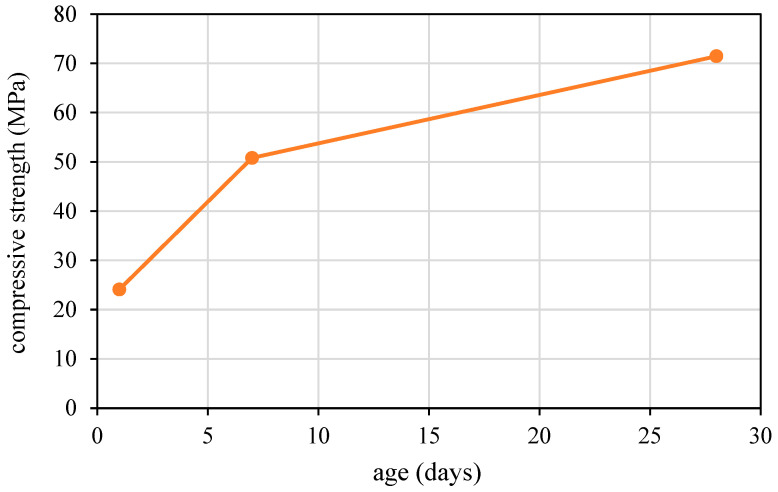
Strength build-up in mortar mixes formulated for 3D printing (Table 1).

**Figure 6:**
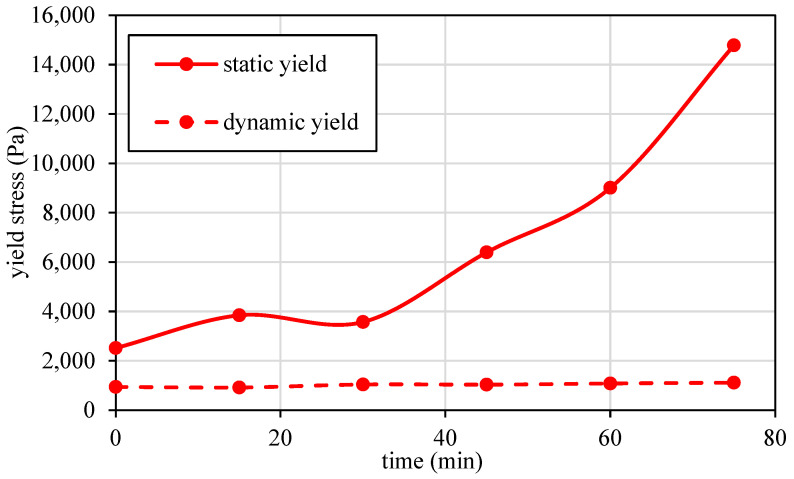
Development of static and dynamic yield stresses of the basic mortar mix designed for 3D printing.

Figure 5 and Figure 6 demonstrate the strength build-up and rheological performance of the basic mortar mixture. The compressive strength, as shown in Figure 5, increases steadily over time, reaching approximately 65 MPa by 28 days, indicating robust strength development suitable for structural applications. The typical rheological performance of the mix in the first 60 minutes, as described in Figure 6, is crucial for 3D printing and includes the following two main stages: (i) a re-flocculation stage, where the rise in the static shear yield stress is due to the physical effect of consolidation and (ii) a structuration stage, which depends on the formation of stronger hydrate bonds [26,55]. Throughout this period, the mix maintains a constant and low-dynamic-yield stress, indicating that reaction products can be readily broken down under dynamic shear conditions.

**Figure 7:**
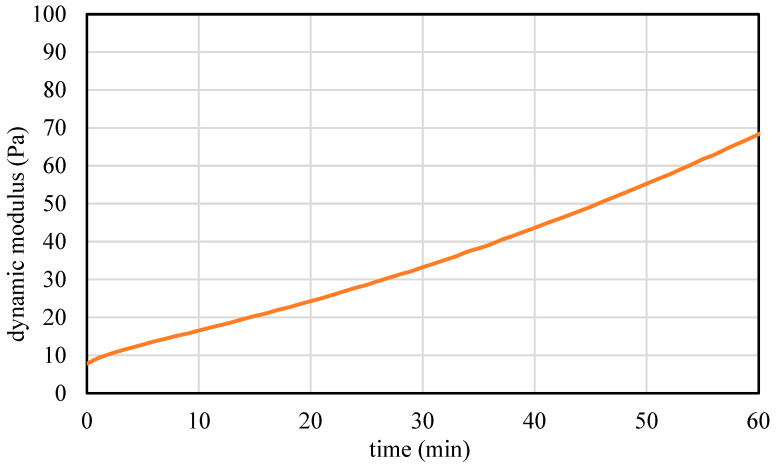
Dynamic modulus of elasticity curves obtained from ultrasonic testing during the first 60 minutes.

**Figure 8:**
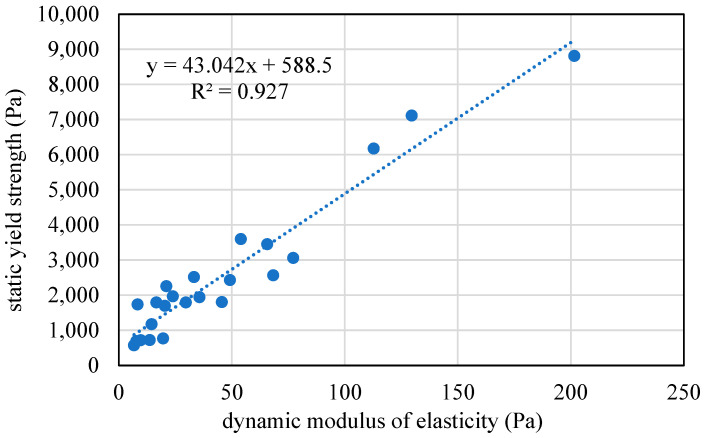
Relation between the dynamic modulus of elasticity and the static yield stress for the mortar mix in Table 1.

Ultrasonic testing results, as shown in Figure 7, illustrate the initial rapid increase in the dynamic modulus of elasticity within the first 60 minutes, which occurred prior to the setting time. This method provides a straightforward and efficient means to assess and optimize the very early-age (60 minutes) properties of various cementitious mixes, ensuring their suitability for 3D printing applications, as demonstrated by the relation between the modulus of elasticity and the static yield stress in Figure 8.

### 3.4. Enhancement of Rheological Properties of the Printing Mix

The early-age rheological behavior of the cement mix suggests that there is room for applying special measures to provide added value by controlling mix properties for 3D printing applications. These can include the use of chemical admixtures, as well as fibers.

#### 3.4.1. Accelerating Admixtures

**Figure 9:**
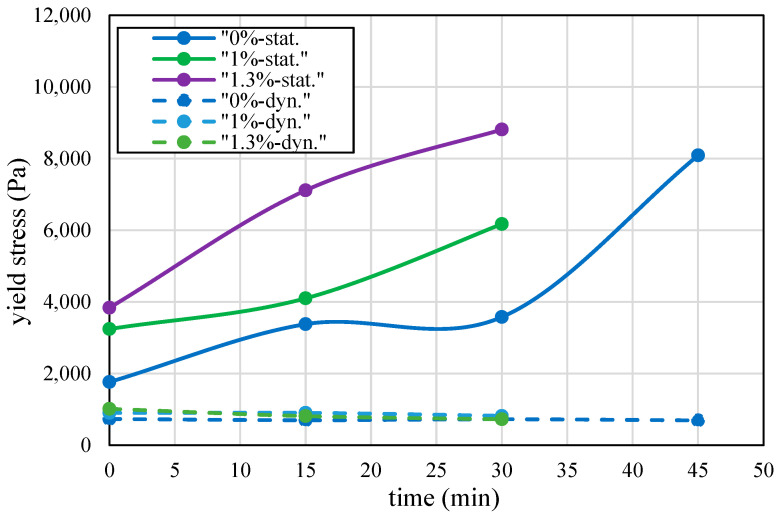
Effect of accelerators on the static and dynamic yield stress values.

One option is using accelerating admixtures, which are applied in the shotcrete industry to accelerate setting, ensuring immediate adherence of the shot mix to surfaces. For this purpose, varying amounts of accelerating admixture (MasterRoc SA 193, an alkali-free set accelerator for sprayed concrete produced by MasterBuilders) were added to the basic mix, corresponding to 1.0 and 1.3 wt.% of the cement content. Rheological characterization, as seen in Figure 9, shows that admixtures effectively enhanced thixotropic behavior by increasing static yield stress during the first hour while maintaining low and constant dynamic yield stress. This improvement in static yield stress indicates that the mix can achieve better structural stability at an early age, without compromising its workability.

**Figure 10:**
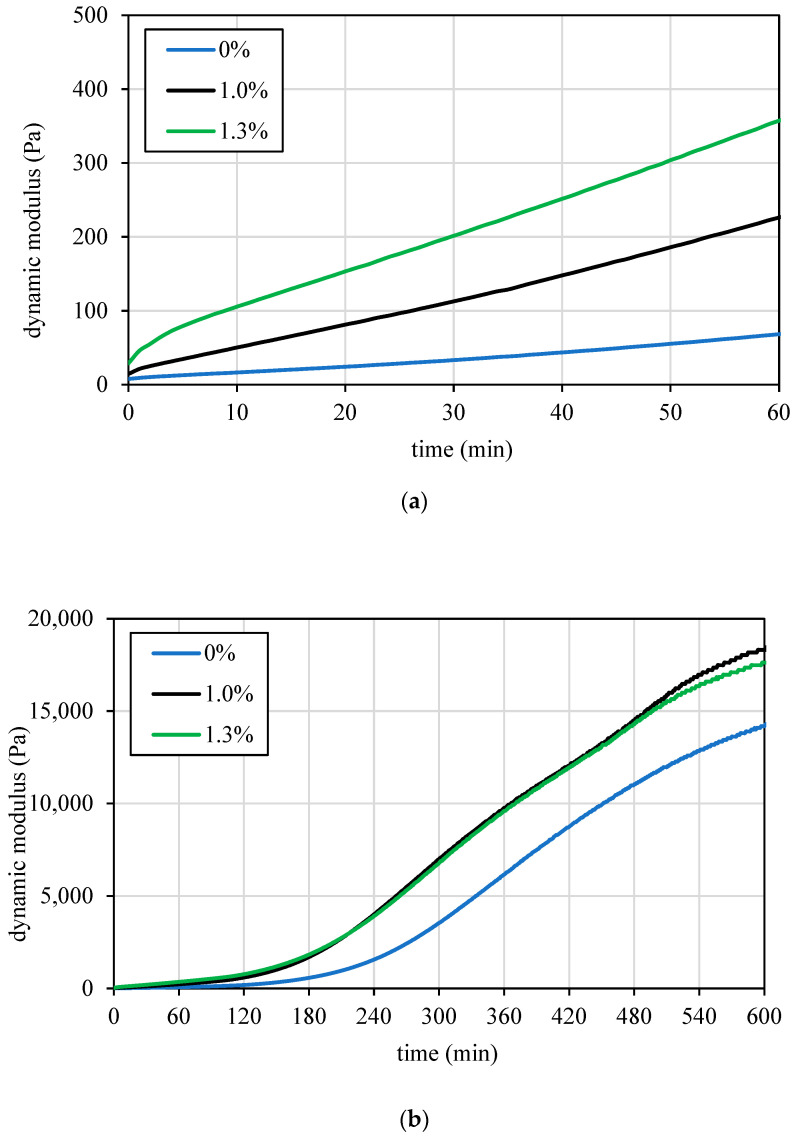
Dynamic modulus of elasticity curves from ultrasonic testing, showing the effect of accelerating admixture on a mix: (**a**) first hour; (**b**) first 10 hours.

Ultrasonic tests further indicate enhanced activity due to the admixture effect, particularly during the first hour (Figure 10a), with diminishing influence over ten hours (Figure 10b). This effect aligns with the concepts of this type of accelerating admixture, influencing aluminate reactions in the initial minutes, providing a quick set and increased early-age strength. Over a longer period, the influence of the admixtures decreases, reflecting the natural progression of the cement’s hydration process and the gradual development of the material’s mechanical properties.

#### 3.4.2. Low-Modulus Fibers

**Figure 11:**
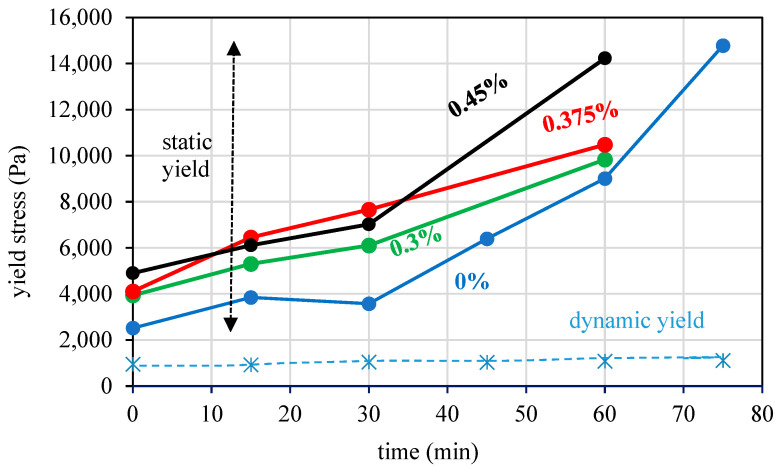
The effect of low-modulus fibers on the rheological behavior of the cementitious mix: black, red, green and blue for the mixes with 0.45%, 0.375%, 0.30% and 0% fiber content by volume for the static yield values, respectively; dashed blue line for the dynamic yield stresses which were practically independent of fiber content.

Another approach to enhance rheological behavior is the addition of low-modulus polymeric fibers, as shown in Figure 11. Fibers significantly increase static yield stress, with minimal effect on dynamic yield stress.

Fiber technology has an advantage over the use of accelerating admixtures because fibers can be more readily premixed, while accelerating admixtures should, preferably, be incorporated by injection into the printing head.

#### 3.4.3. Mature Properties of the Printed Mix

The effects of these rheology-enhancing technologies on early-age rheological properties have no consequences with respect to the mature properties, as determined by compressive strength tests of 50 mm cubes cast from the printing mix and cured in standard conditions. For the various accelerator contents, they ranged from 23 to 26 MPa on 1 day, 39 to 45 MPa after 7 days and 65 to 70 MPa after 28 days.

**Figure 12:**
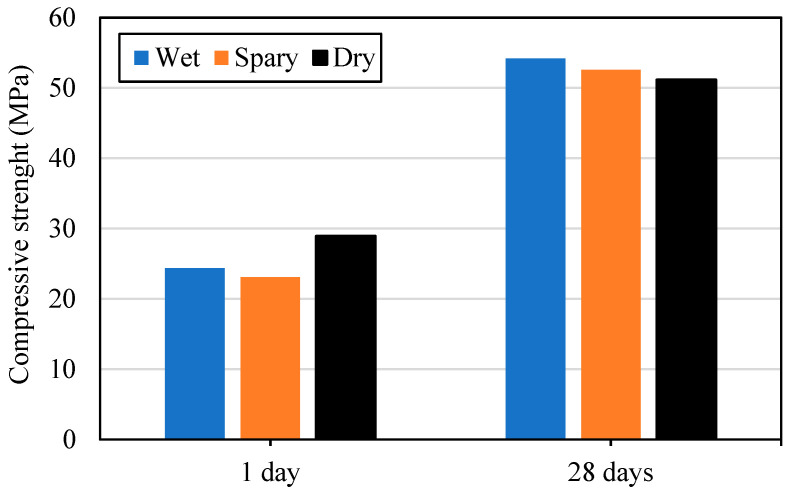
Effect of curing on the strength of the mix.

For these compositions of mixes, there was no practical influence of the curing regime on the strength achieved at various ages, as shown in Figure 12. This might be explained by the relatively low w/c ratio, where even minimal hydration is sufficient to seal the mix and keep the water inside available for continued hydration.

## 4. Manufacturing Parameters

Controlling the manufacturing parameters according to the rheological properties of the mix is crucial for a successful 3D printing process, ensuring buildability and dimensional stability. To achieve this, we rely on an analytical model developed by Kruger et al. [54]. This model, grounded in the analysis of rheometer test data, provides a comprehensive understanding of layer stability and informs the overall design strategy.

### 4.1. Buildability Considerations

According to Kruger et al. [54], the shear stress at the bottom layer can be calculated as follows (Equation (1)):

(1) $$\tau =\frac{\rho gh}{2F_{AR}},$$

where

- τ is the shear stress (Pa);
- *ρ* is the density of the material (kg/m^3^);
- *g* is the gravitational acceleration (m/s^2^);
- *h* is the element height (m); and
- *F_AR_* is a strength correction factor that accounts for confinement due to the layer aspect ratio (h/w).

By applying this model, we can gain control over layer stability during the 3D printing process. This is achieved by ensuring that the shear stress applied upon the layer is smaller than the material’s static yield stress throughout the whole printing process. This understanding enables the adjustment of manufacturing parameters, such as printing speed, layer height, and extrusion rate, in accordance with the rheological properties of the mix. Consequently, this ensures the stability and structural integrity of the printed layers, leading to a more efficient and reliable 3D printing process.

However, a more nuanced approach is necessary for complex geometries where the cross-section varies throughout the specimen. Mogra et al. [46] extended Kruger et al.‘s [54] work by developing an analytical model to optimize the buildability of a 3D-printed element. This model assumes that the loading stress from the piled-up layers develops incrementally, with the self-weight of a deposited layer considered only after the entire layer has been added.

**Figure 13:**
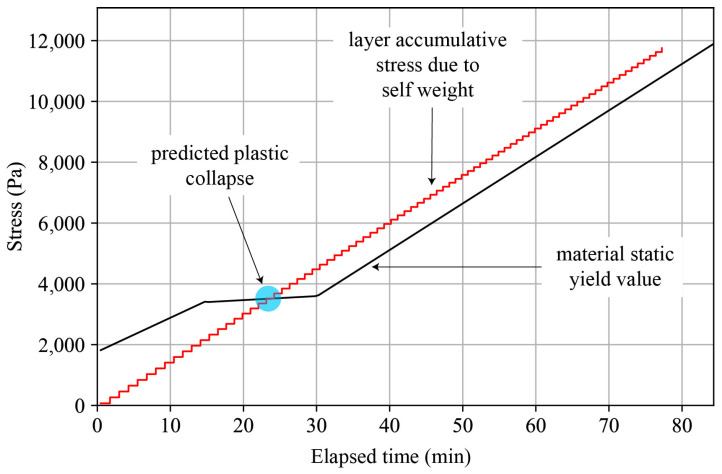
Schematic plot predicting plastic collapse during the printing process. The red line represents the cumulative stress due to self-weight on a single layer, while the black line indicates the material’s static yield stress value. If the cumulative stress surpasses the static yield value, plastic collapse is predicted, as shown by the intersection marked with a blue circle.

Similarly, we account for the self-weight of each layer as it is deposited, which better reflects a real-world scenario where failure might occur at the bottom of any layer, and the weight above the failure point includes the layer itself. Figure 13 describes a schematic representation of such an approach, where the red line represents the shear stress experienced by a given layer and the black line represents the material static yield stress obtained from the rheological test.

### 4.2. Interactive Plug-In for Optimized Manufacturing Process

To create a streamlined process where the optimization of a manufacturing process can be easily controlled, an interactive Python plug-in for Grasshopper® software (Version 1.0.0007)as developed. Grasshopper® is commonly used by designers and architects for the development of parametric designs, and it is widely applied in the creation of designs for 3D printing processes. This tool allows the designed structural element to be readily tested for buildability, facilitating an iterative optimization process.

**Figure 14:**
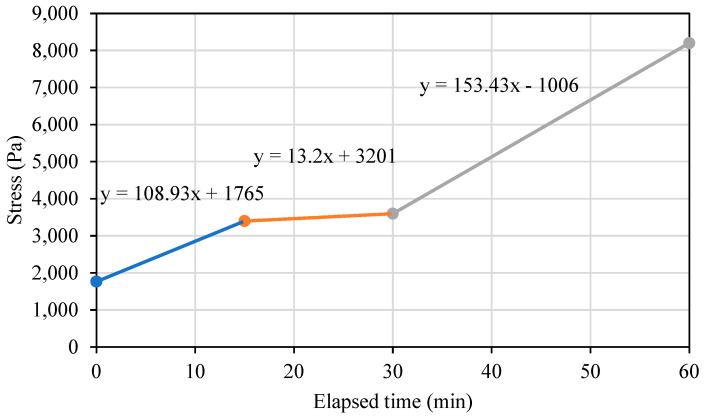
Linear functions fitted to the static yield evolution plots for a basic mortar mixture over the following three distinct time periods: 0 to 15 minutes, 15 to 30 minutes, and 30 minutes forward.

To integrate the material static yield stress into the plug-in, linear functions are fitted to each tested material based on the following three time periods: 0 to 15 minutes, 15 to 30 minutes, and 30 minutes onward, as illustrated in Figure 14 for the basic mortar mixture. The intersection points (Pa) and slope (Pa/min) are calculated for each linear function, creating a continuous function of the static yield stress build-up over time for each material.

The plug-in outputs a plot predicting the plastic collapse of each layer individually after its printing process begins. Each layer is represented in a step plot that describes the stress it will experience throughout the entire printing process. The bottom layer’s plot is the longest, corresponding to the entire printing time, while the top layer’s plot is the shortest, reflecting only the print time of the top layer. If the cumulative stress on the layers surpasses the plot describing the material’s static yield stress, the specimen is predicted to fail due to plastic collapse.

The parameters controlled by the user include the printing curves, layer width, layer height, density of the fresh material, printing velocity, and rheological properties of the material. Once a parameter is changed, the plug-in instantaneously outputs a plot indicating whether plastic collapse is predicted, allowing for rapid iteration and optimization of the process based on all the parameters described above.

**Figure 15:**
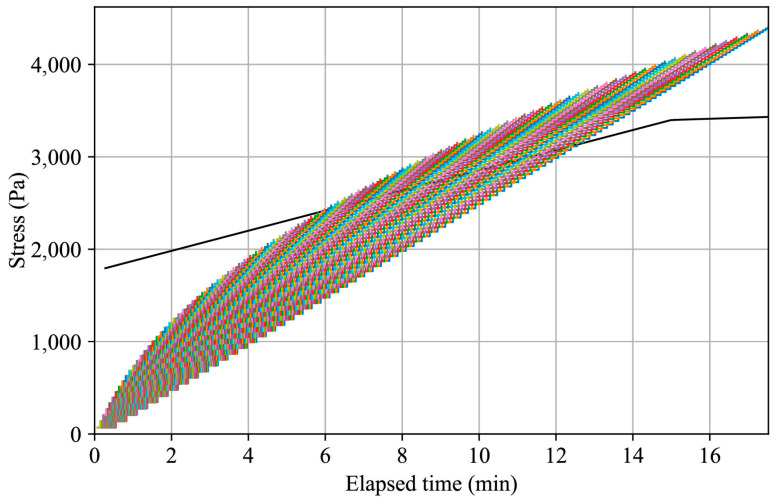
Stress evolution over time for a tapered cylinder with a bottom radius of 400 mm, a top radius of 100 mm, and a height of 1000 mm printed with a basic mortar mixture (density of 2130 kg/m^3^) using a layer height of 10 mm and layer width of 20 mm at a printing velocity of 150 mm/s. The colorful step plot shows the cumulative stress on each layer, indicating whether the layers are predicted to experience plastic collapse based on the material’s static yield stress.

For example, consider a tapered cylinder with a bottom radius of 400 mm, a top radius of 100 mm, and a height of 1000 mm. The material used for the printing process is the basic mortar mixture described in Table 1, with a density of 2130 kg/m^3^. The selected layer height and width for the process are 10 mm and 20 mm, respectively. The output plot of the plug-in with a printing velocity of 150 mm/s is presented in Figure 15. According to the results, the specimen is predicted to collapse. The collapse will take place in the bottom layers, as evidenced by the surpassing of the material’s static yield stress values. Theoretically, the top layers, experiencing less shear stress due to the tapering and reduced weight, are not expected to collapse. This suggests that a lower printing velocity is required to prevent the collapse of the specimen.

## 5. Design Optimization

Our holistic framework is applied in this study to the design and manufacturing of a post-tensioned beam. Post-tensioning allows the challenge of integrating reinforcement into printed parts to be circumvented, which is a topic of ongoing research efforts. We follow the conceptual idea of Vantyghem et al. [38], who used post-tensioning to join several printed segments and assemble a continuous girder that can sustain realistic external loads in bending. In the current study, we designed and fabricated a 4 m long beam by joining two 2 m long printed segments.

**Figure 16:**
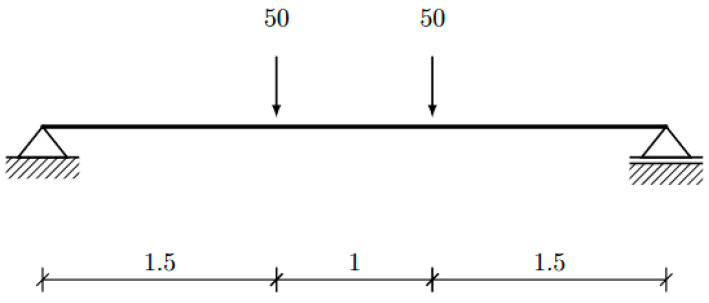
The 4-point bending setup of the beam to be optimized and manufactured.

We designed the beam for a simple four-point bending setup, as depicted in Figure 16. The beam should sustain its own self-weight and two external point loads. For design optimization, we assumed a magnitude of 50 kN for each of the loads. We applied a shape optimization procedure, where the shape of the beam’s cross-section is optimized using an explicit spline-based parameterization of the cross-section shape [56], following the formulation proposed by [46].

Minimize: $f^{T}u$

subject to: $\sum_{i=1}^{n}A_{i}\left(s\right)h-V_{0}\;\leq 0$

$$\bar{y_{j}}\geq \bar{y_{0}}\;j=1,\ldots ,n\left(n_{p}-1\right)$$

$$d_{i}\leq d_{0}\;i=1,\ldots ,n$$

$$s_{min,k}\leq s_{k}\leq s_{max,k}\;k=1,\ldots ,m$$

with:

 K(s)u=f

The problem formulation is repeated here for completeness. Here, the vector of design variables **s** contains the coordinates of the control points of each layer. The shape of the beam is represented by the collection of layer-wise cross-sections. The number of layers in the computational model is *n*, and the number of control points that parameterize each cross-section is *n_p_*. The objective function to be minimized is the compliance under and the external loads **f**, which contain self-weight (which depends on the design variables) and two-point loads. The displacements **u** are found during each design iteration by solving the linear elastic equilibrium **K**(**s**)**u = f**, where the stiffness matrix **K** is integrated according to the exact cross-section shape of each layer.

We impose a constraint on the volume, as we aim to improve the overall stiffness-to-weight efficiency of the beam in comparison to simple rectangular members. In the volume constraint, *A_i_* is the area of the *i^th^* cross-section; *h* is the height of the layer, which is uniform in the current study; and *V*_0_ is the allowable volume of material. Buildability is considered as part of the design optimization problem in the form of the following geometric constraints: we enforce an overlap between consecutive layers by limiting the distance *d_i_* between consecutive centroids to *d*_0_ = 7.5 mm. Finally, the shape of each cross-section is controlled by box constraints that are imposed on all *m* design variables. In order to avoid self-intersection of B-spline curves during optimization, the difference between the *y* coordinates of adjacent control points is kept positive by requiring the gap between *y* coordinates to be greater than a predefined value (*y*_0_, set as 0.05 m in the example). Optimization was executed using an in-house MATLAB (Version R2023a) code with the internal optimization solver fmincon [57]. The cross-section shape is bounded within a square of 400 mm side length. The volume is constrained to 50% of the volume of the bounding box. Exploiting the symmetry of the problem, we optimize only one half of the beam consisting of 100 layers, each of them 20 mm high. The optimization assumes that the cross-section is eventually solid, meaning that an additional either casting or grouting step is required (as in [38]) or that printed infill material will be added. In the current study, we resort to the second option because it opens the opportunity to demonstrate the interaction between structural design and physical buildability, as discussed in the next section.

**Figure 17:**
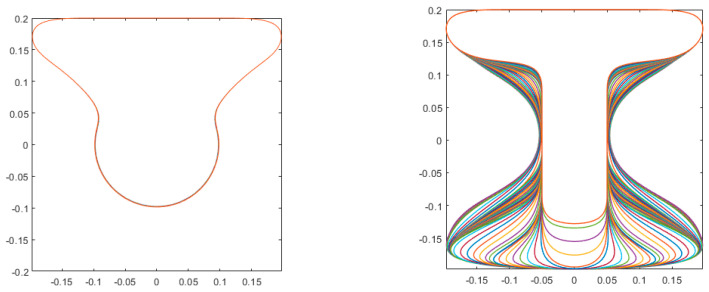
Shape optimization of the beam using spline parameterization of layer-wise cross sections. **Left**: initial design; **Right**: 2D view of the optimized design, showing all 100 cross sections. Each colored line represents a single layer.

**Figure 18:**
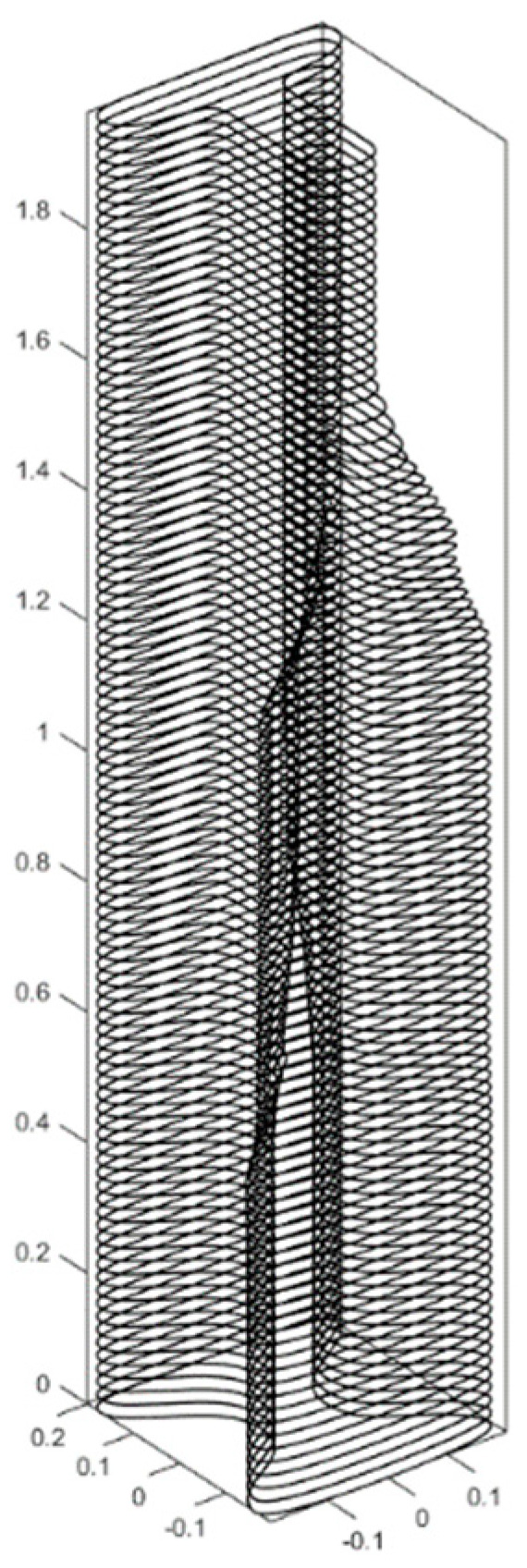
Shape optimization of the beam using spline parameterization of layer-wise cross sections. Three-dimensional view of the optimized design, showing all 100 cross sections in the intended printing position of each symmetric half of the beam.

The initial design is a uniform cross-section, as shown in Figure 17 (left). The upper edge of the square domain is intended for positioning of the loads; hence, it is predefined and not optimized. The optimization generates the design depicted in Figure 17 (right) and 18. The shape resembles a T-section near the supports that gradually and smoothly converts to an I-section where the maximal bending moments occur. The resulting geometry, as shown in Figure 18, is the input for a final design-for-manufacturing step, where we simultaneously determine the tool path for printing, the geometry of post-tensioning cables, and the forces in the cables that adequately balance the external loads. This is described in the next section.

## 6. Integration in Digital Blueprint

**Figure 19:**
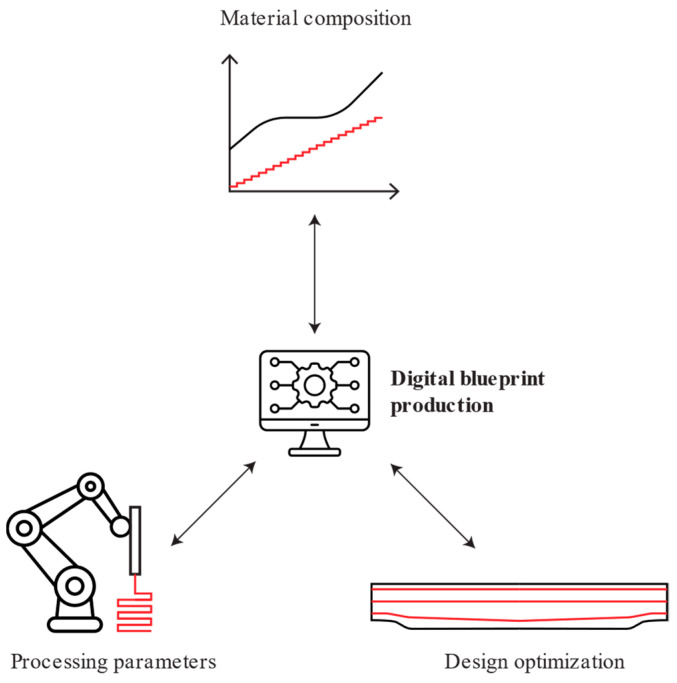
The digital blueprint production process at the intersection of material characteristics, processing parameters, and design optimization.

The digital blueprint integration sits at the junction of material characteristics, manufacturing parameters, and the optimized design. Here, an integration of the three abovementioned factors takes place, facilitated by a computational workflow, as described in Figure 19. We demonstrate our approach through the case study of the optimized beam described in previous sections.

**Figure 20:**
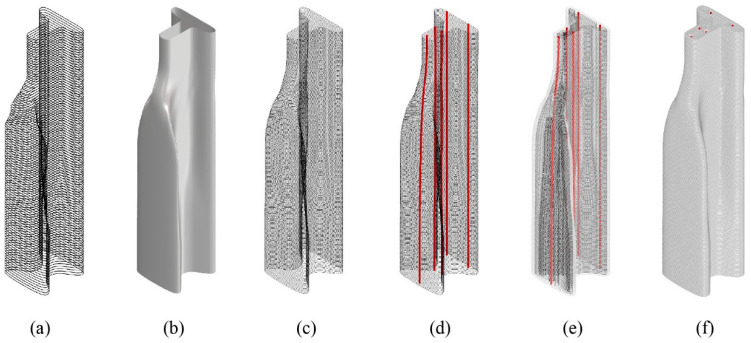
Step-by-step illustration of the digital blueprint production process for the designed half-beam curves. (**a**) Initial design of the half-beam curves; (**b**) creation of a smooth surface from the curves; (**c**) slicing the surface into the desired layer height; (**d**) placement of post-tensioning cables (in red); (**e**) iterative production of the infill pattern according to cable locations (in red); (**f**) visualized final 3D model.

The printing tool path was parametrically and iteratively generated within the Grasshopper^®^ environment, with its stages described in Figure 20. First, the optimized half-beam cross-sections were used to create a smooth surface. Then, the surface was sliced to the desired layer height of the printed specimen, with a 10 mm layer height chosen for this study. Subsequently, the post-tensioning cables were placed, considering the maximum bending radius of the post-tensioning cable. Following their placement, the coordinates of each cable in each layer were used to calculate the performance of the full beam after post-tensioning.

After achieving satisfactory results in performance calculation, the infill layers of the half-beam were produced. For each cable, measuring 12 mm in diameter, a 40 mm diameter circle was placed at the cable coordinates to allow for the insertion of a duct for cable placement. An enlarged diameter was chosen to compensate for the narrowing from the initial computational design to the printed layer width. Each exterior shell layer was then offset according to the desired layer width to create the first infill layer, and the circles for the cables were subtracted from the infill curve, creating a conduit for the cables. To ensure approximately 80% filling of the beam, a second infill layer was created, offset from the first infill layer. Outliers and artifacts from the offsetting procedure were filtered to ensure that only the desired infill pattern remained, generating the final printing path.

We note that the design of the infill, the cable geometry, the cable force, and the buildability are all inter-related. Hence, several design iterations were performed to find an adequate combination of infill pattern and prestressing forces that meets the requirements of both structural design and buildability. The final prestressing consists of five cables—one curved cable that follows the bottom part of the beam, tensioned to 130 kN; two straight cables with a small eccentricity, tensioned to 80 kN each; and two straight cables that follow the top part of the beam, tensioned to 20 kN each.

A basic mortar mixture, as described in Table 1, was chosen to validate the integration method and print the optimized beam. To select the appropriate processing parameters, the following initial parameters were set: a layer height of 10 mm; a layer width of 25 mm; a density of 2130 kg/m3, which remained static for this study; and a printing velocity that was dynamically changed according to the results.

**Figure 21:**
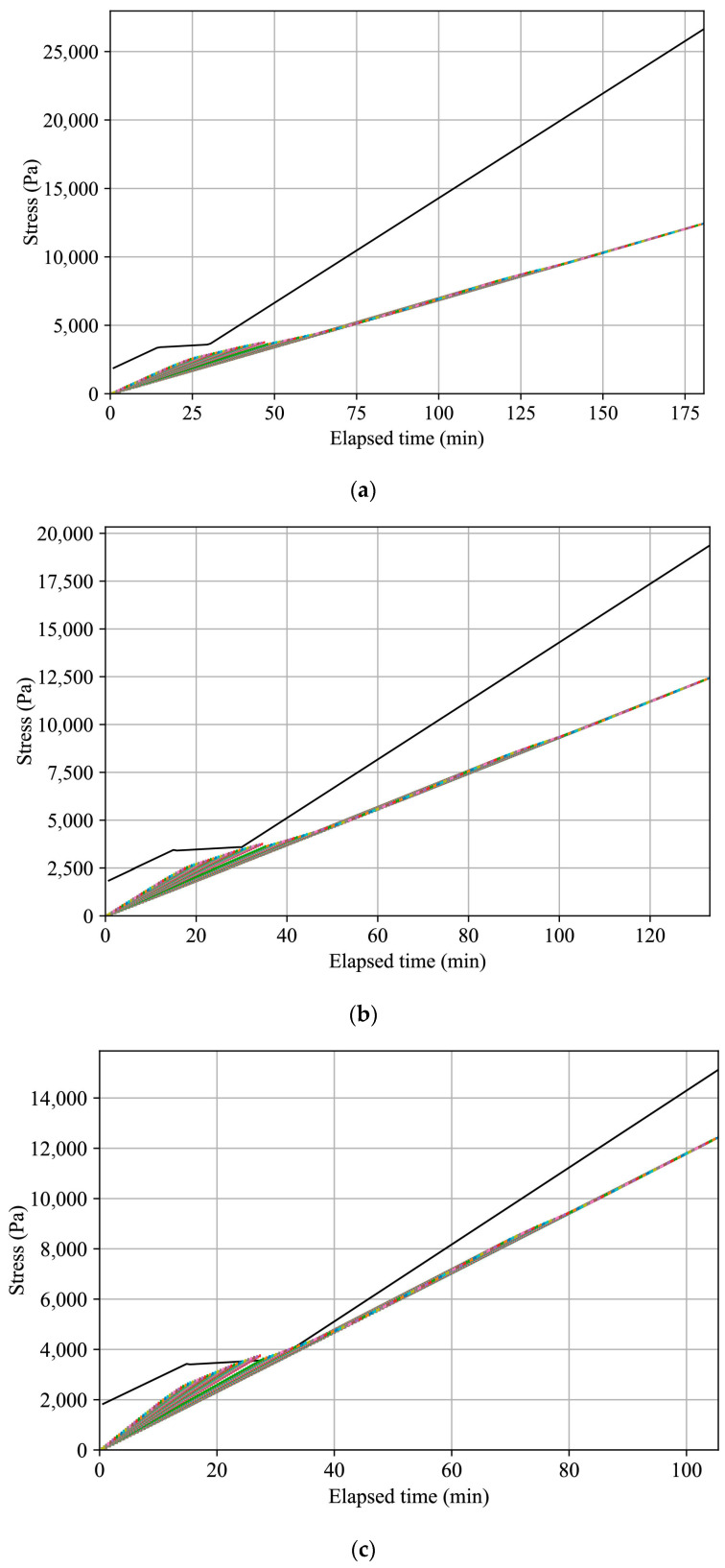
Plots of plastic collapse prediction of half-beam print path based on the material static yield evolution of the basic mortar mixture with varying printing velocities ((**a**) 70 mm/s; (**b**) 95 mm/s; (**c**) 120 mm/s). The black plot describes the material’s static yield stress as a function of elapsed time. The colored step plot describes the cumulative stress evolution in each printed layer.

Adjustments were made to the printing velocity to ensure the cumulative stress on the printed layers would not surpass the material’s static yield stress. Figure 21 shows the output plot of the plug-in as a step plot of the cumulative stress evolution within the printed half-beam layers at the following three printing velocities: 70, 95, and 120 mm/s. The results suggest the optimal printing velocity is 95 mm/s. While 70 mm/s does not lead to plastic collapse, as predicted by the plug-in, there is a margin for improvement and reduction in the printing time. In contrast, with a printing velocity of 120 mm/s, plastic collapse is expected.

In the integration process for manufacturing the optimized beam, the locations of the post-tensioning cables, infill pattern, and printing velocity were the dynamic parameters optimized according to a designated material composition. However, our approach is flexible and allows for changes in any component of the integration process, which influences the entire manufacturing workflow. For instance, if the design of the specimen must remain unaltered but a shorter printing time is required to meet specific manufacturing deadlines, adjustments can be made to the material composition to achieve these requirements. This adaptability ensures that the overall process can be fine-tuned to meet various design and production goals efficiently.

## 7. Manufacturing the Optimized Beam

### 7.1. Three-Dimensional Printing of the Half-Beams

The printing process was carried out using a 3D printing robotic-arm setup. The printing setup comprised a KUKA KR50 (KUKA Deutschland GmbH, Augsburg, Germany) industrial robotic arm coupled with a MAI^®^ MULTIMIX-3D (MAI^®^ International GmbH, Feistritz/Drau, Austria) mortar pump. A 10-meter hose was used to transport the material. A metal rod was fixed to the end of the robot’s kinematic system, serving as the printhead, with a 12.5 mm diameter round nozzle. Prior to printing, the hose was lubricated with a mixture of kaolinite clay and water to reduce friction.

**Figure 22:**
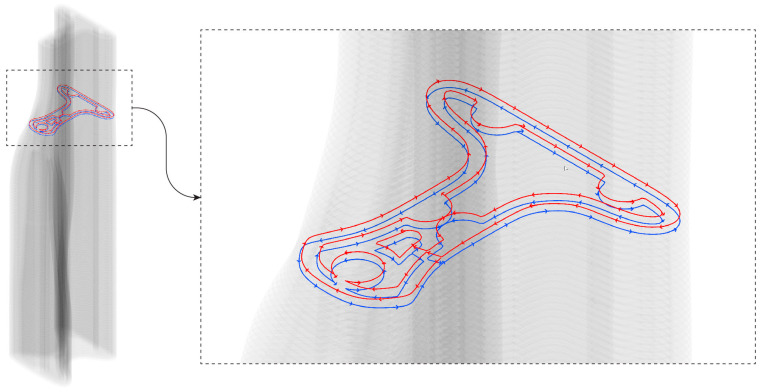
Illustration of the continuous printing path for the half-beams, showing the flipped printing direction of two alternating layers in two different colors, including the infill pattern.

Two half-beams were printed according to the designed paths resulting from the process described in Section 6. The setup used in this research allows only for a continuous printing process, which requires proper preparation of the print path to ensure the printed model matches the design model. To achieve this, a seam line was required, positioned in the lower-right part of the half-beam to align with the location of the innermost infill layer. To create a continuous path, the printing direction of every other layer was flipped, and the subsequent layer was connected to it at the seam point, as seen in Figure 22. The curves were then divided into 5 mm resolution points that were converted into planes for robot positions. The Kuka PRC plug-in for Grasshopper was used to create the robot code, including the chosen velocity of 95 mm/s.

**Figure 23:**
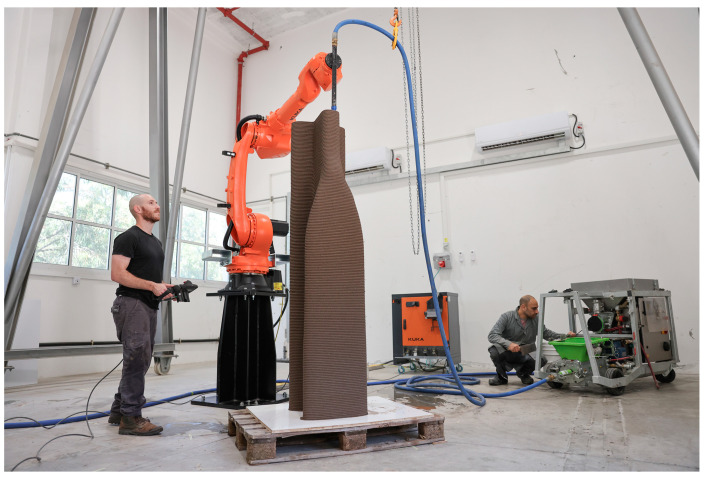
The 3D printing process of the first half-beam using a robotic cell setup.

**Figure 24:**
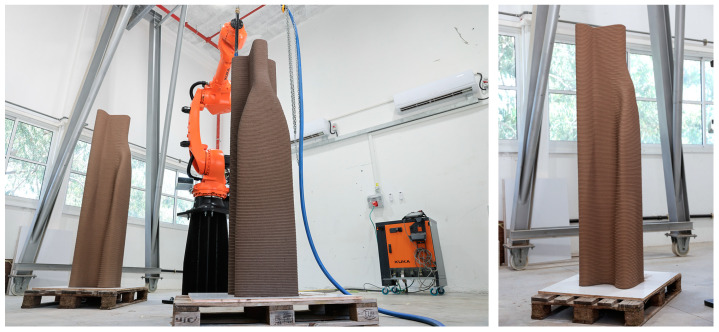
(**Left**) Printing the second half-beam. (**Right**) The completed half-beam, demonstrating a successful execution of the printing process over two consecutive days.

The printing of the two 2 m half-beams was completed without any signs of obvious deformation or plastic collapse, as depicted in Figure 23 and Figure 24, confirming the efficacy of the buildability algorithm used for this specimen. The manufacturing parameters are detailed in Table 5. The continuous printing process was carried out over two consecutive days with the KUKA KR50 industrial robotic arm and MAI^®^ MULTIMIX-3D mortar pump, demonstrating the robustness of the prepared printing path and the material selection. The application of the buildability algorithm, which considered the material properties in their fresh state, i.e., static yield stress evolution and printing velocity, proved to be efficient in predicting the structural integrity of the printed layers in fresh state for large elements. The absence of plastic collapse and deformation indicates that the optimized parameters, including the printing velocity of 95 mm/sec, were well adapted to the process. This successful outcome validates the computational workflow and algorithm and underscores the importance of planning and parameter selection in additive manufacturing processes.

### 7.2. Quality Monitoring of the Two Half-Beams

Quality monitoring of the two half-beams by evaluating the geometric accuracy of the printed model in relation to the computational model was set out as part of the manufacturing process. This step is crucial in the manufacturing of structural elements that require precision. For this purpose, a Trimble X7 (Trimble Inc., Sunnyvale, CA, USA) light detection and ranging (LiDAR) scanner was used. The LiDAR scanner uses emitted laser pulses to accurately represent points in x, y, and z coordinates, creating a digital representation via a point cloud of the scanned specimen.

**Figure 25:**
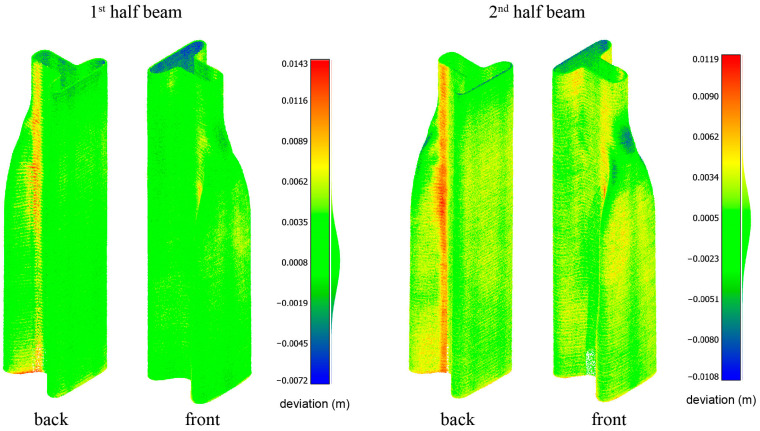
Geometric accuracy evaluation of the printed half-beams. The color maps show the deviation of the scanned half-beams from the computational model. The first half-beam (**left**) and the second half-beam (**right**) are displayed from both back and front views. The color scale represents the distance deviation in meters.

The half-beams were positioned upright, as seen in Figure 24, and scanned from four points to create a complete image of the beam. Registration was performed automatically using Trimble Perspective (Version 3.1.1.1878) software, with an accuracy of under 1 mm between scans. The scans were then transferred to CloudCompare (Version 2.13.2), an open-source point-cloud processing software. A filtering procedure created a 2 mm distance point-cloud model of each half-beam, containing approximately 850,000 points each. A registration tool aligned the filtered point cloud with a digital mesh produced using Grasshopper^®^, representing the desired layer width (see Figure 20f). A distance measuring tool was then used to measure the distance between the point cloud of the specimens and the digital mesh model. The results of the analysis are presented in Figure 25.

The results of the distance analysis indicate a high level of accuracy between the printed specimens and the computational models. The first printed specimen exhibited an average deviation of + 0.0008 m from the computational model, with most deviations falling between − 0.002 and + 0.005 m. The second printed specimen showed an average deviation of + 0.0005 m, with deviations primarily ranging from − 0.005 to + 0.005 m. Both specimens displayed a pronounced positive deviation in the seam area, visible as a red vertical region, which is more prominent in the second specimen. This anomaly is attributed to the change in direction and height during layer transitions while the printhead continues extruding material. During this brief period, over-extrusion occurs, resulting in excess material deposition. This issue could be mitigated in future prints by designing a tool path that compensates for this phenomenon, possibly by retracting the extruder slightly at the end and beginning of each layer. Alternatively, using a printhead capable of pausing extrusion during layer transitions could prevent this over-extrusion. Additionally, some negative deviations were observed in areas with significant overhang angles. These are second-order effects, and in order to address them, there is a need for additional study considering viscoelastic effects.

## 8. Further Optimization of the Printing Process by Rheological Property Enhancement

Building upon the digital blueprint integration process described in Section 6, the method was utilized to demonstrate a theoretical optimization of a printing process using various materials developed in Section 3. These modifications include the addition of accelerators or fibers to the basic mortar mix, with the aim of improving static yield stress and thixotropic behavior, thereby reducing the overall printing time of the beam. This section showcases the potential of our approach in using the same printing path applied for the final printing of the half-beams, optimizing the printing velocity according to the selected materials.

**Figure 26:**
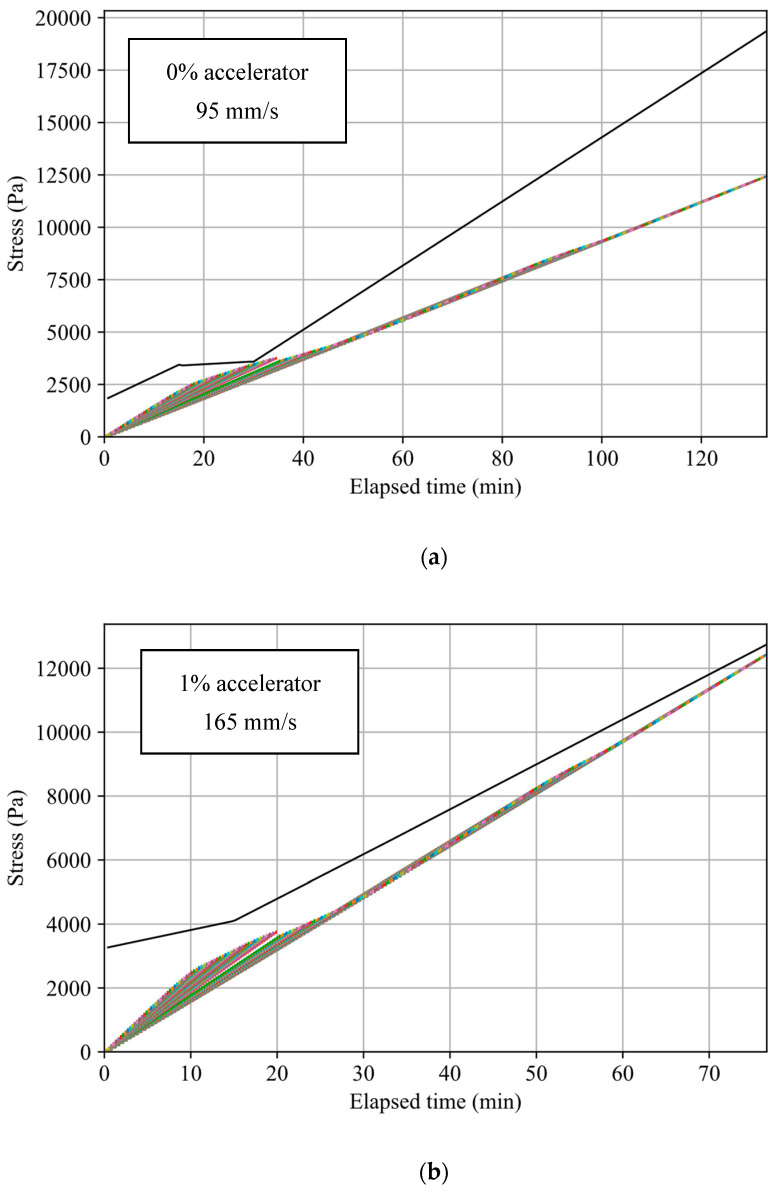

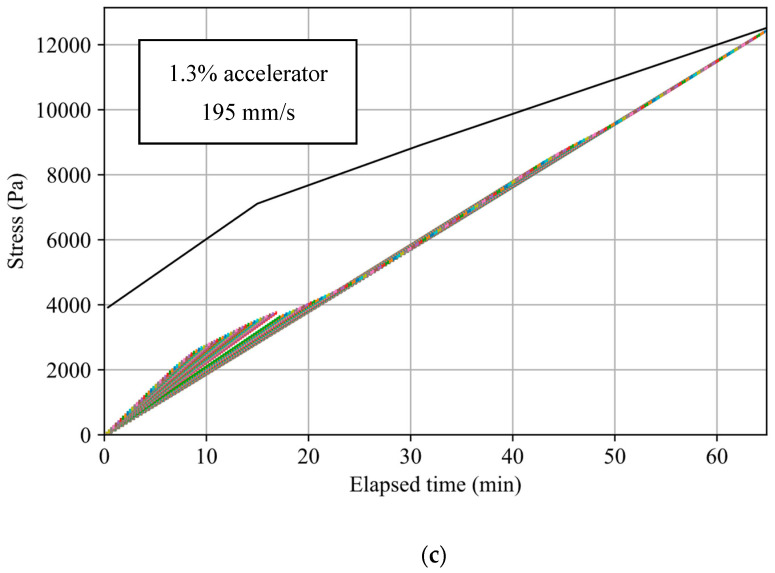
Optimized plots of layer stress buildup based on accelerator-enhanced mixtures’ static yield stress evolution with varying printing velocities ((**a**) 0% accelerator; (**b**) 1% accelerator; (**c**) 1.3% accelerator). The black plot describes the material’s static yield stress as a function of elapsed time. The colorful step plot shows the cumulative stress in each layer, indicating whether the layers are predicted to experience plastic collapse based on the material’s static yield stress.

The mixture with accelerator static yield values described in Figure 9 was used to test the effect of adding an accelerator on the printing velocity and consequent printing time. The static yield plot was fitted to linear functions, as described in Section 6. A linear interpolation of the plot was performed between 30 and 60 minutes due to limitations in testing, as the mixtures with accelerator hardened within this time. The results of the optimized printing speed for each mixture are described in Figure 26 and Table 6. The results demonstrate an improvement in printing time with an increasing accelerator percentage in the mix.

**Figure 27:**
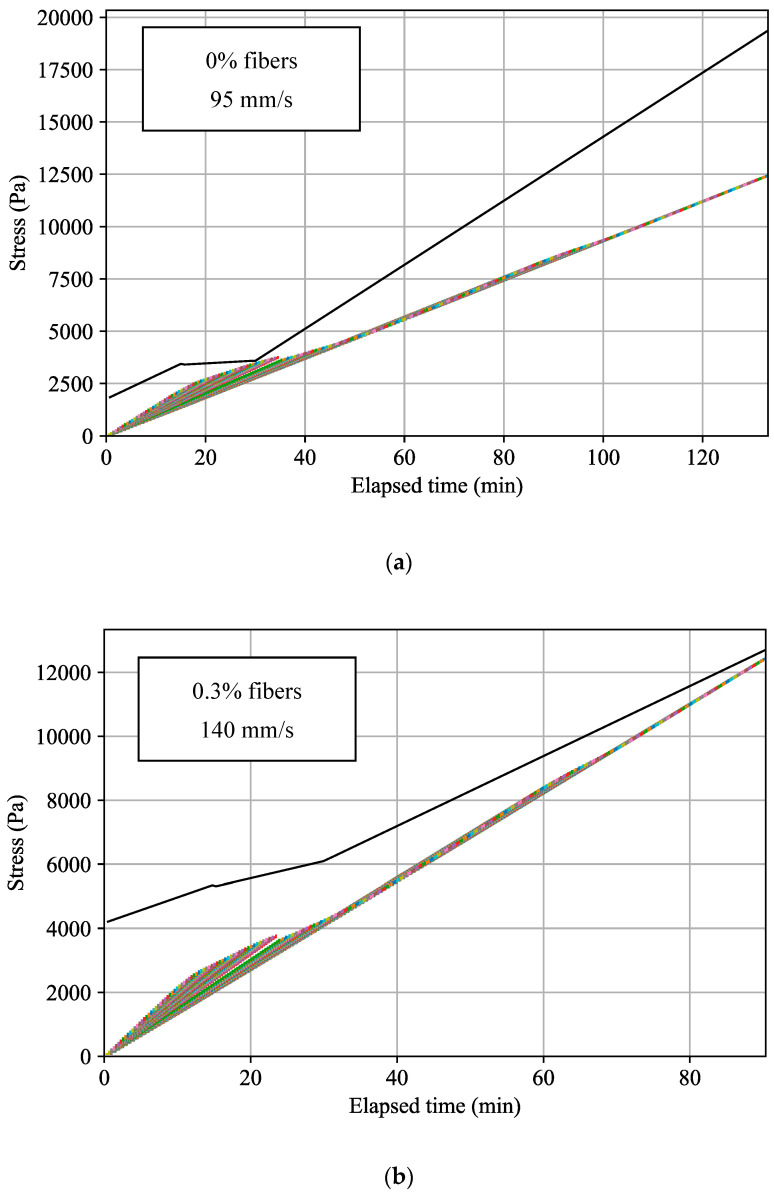

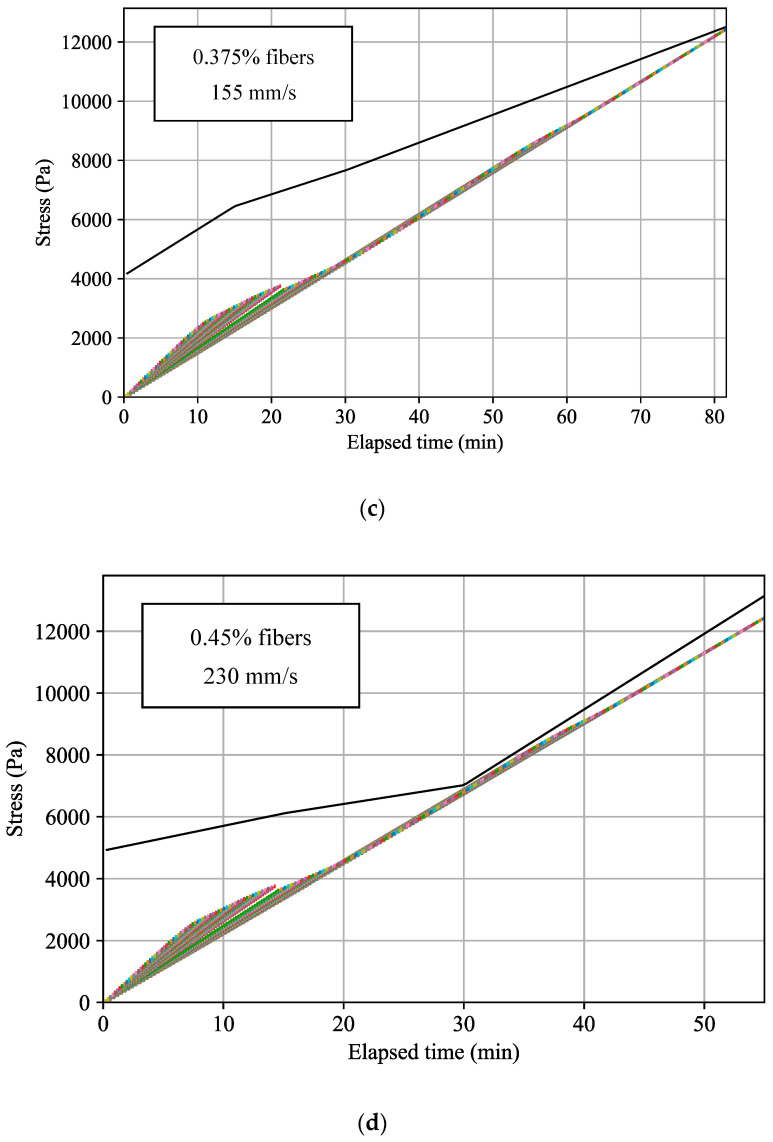
Optimized plots of layer stress buildup based on fiber-enhanced mixtures’ static yield stress evolution with varying printing velocities ((**a**) 0% fibers; (**b**) 0.3% fibers; (**c**) 0.375% fibers; (**d**) 0.45% fibers). The black plot describes the material’s static yield stress as a function of elapsed time. The colorful step plot shows the cumulative stress in each layer, indicating whether the layers are predicted to experience plastic collapse based on the material’s static yield stress.

In addition, the mixtures with fibers described in Figure 11 were used to test the effect of the addition of fibers on the printing velocity. The static yield plot was fitted to linear functions, as described in Section 6. The results of the optimized printing velocity for each mixture are illustrated in Figure 27 and Table 7. The results suggest an improvement in printing time with increasing fiber content in the mix.

The simulation results for the rheologically enhanced mixtures indicate significant improvements in printing velocity and overall production times, demonstrating the effectiveness of the holistic approach for optimizing the printing process. This improvement is primarily due to the enhanced static yield stress and thixotropic behavior of the mixtures, which allow for faster layer deposition, ultimately leading to more efficient and streamlined production. Additionally, the analysis of the manufacturing time based on the rheological properties suggests that a 0.45% fiber mixture is ideal in terms of production time for the demonstrated beam. This result is advantageous from a manufacturing perspective, as it reduces the risk of material blockage in the transportation system when accelerator technology is being used, which can lead to costly implications for the printing setup.

The degrees of freedom afforded by this method allow for the optimization of several aspects of the process according to the specific requirements of the element and the constraints of the manufacturing environment. This flexibility enables the tailoring of parameters to achieve the best possible outcomes, ensuring both efficiency and quality in additive manufacturing of construction elements.

## 9. Conclusions

The holistic approach presented in this paper demonstrates how the various components associated with 3D printing can be integrated to provide a platform that can enable the design and production of optimized building components. It highlights the notion that 3D printing should not be viewed as merely another manufacturing technology associated only with advantages of automation and savings of formwork. Rather, it should be addressed as an integrative platform, providing tools to architects and engineers to enable the design and production of innovative construction components and structures. This can be facilitated by leveraging the degrees of freedom provided by this platform.

The research described in this paper identifies the three major components of this platform—materials, design, and production—and demonstrates that relevant quantitative tools can be developed based on multidisciplinary concepts, covering rheology of materials, structural optimization, design simulations, and robotic production, which can be integrated into a platform enabling the design and production of optimized components, targeting a range of performances. In the current study, the use of this platform was demonstrated to achieve the optimization of architectural and structural performance in the case of a post-tensioned beam to be assembled from two printed halves.

The case study demonstrates the integration of a mix design tailored for optimal rheological properties, with a structurally optimized beam that improves stiffness-to-weight efficiency. The use of a Grasshopper plug-in, which evaluates printing failure based on plastic collapse related to the static yield stress of the mix, enabled the optimization of the printing velocity to align with the specific material–design interaction. This approach ensured an efficient printing process, as validated by the successful printing and dimensional evaluation of the two optimized half-beams. Furthermore, this study demonstrates the potential for further optimization through the use of rheologically enhanced mixtures, underscoring the versatility of a holistic approach in additive manufacturing.

Such a holistic approach exemplifies the need for a synergetic model capable of combining design, material, and production needs to generate significant economy of material, labor, and time in construction.

## Figures and Tables

**Table 1:** Composition of the basic mortar mix for printing in units of kg per m^3^ of mortar.

Component	kg/m^3^
Cement, CEM I	459.3
Kaolinite clay	98.5
Microsilica	98.5
Sand	1221.3
Water	238.6
Polycarboxylate admixture (HTC 698)	13.2
Unit weight	2129.4

**Table 2:** Mineralogical composition of Type I 52.5 cement.

Compound	Weight%
C3S	54.1
C2S	19.55
C3A (Cubic)	5.03
C3A (Ortho.)	1.86
C3A (Total)	6.89
C4AF	12.17
CSH0.5	1.84
CH	0.62
CC	4.54
Aphthitalite	0.59

**Table 3:** Mineralogical composition of chocolate clay.

Phase	Weight%
Kaolinite	76.8
Quartz	12.7
Calcite	1.4
Ivsite	3.9
Picromerite	3.1
Orthoclase	2.1

**Table 4:** Statistical parameters of the cements and mineral admixtures used in this study.

Admixture	Dx (10), μm	Dx (50), μm	Dx (90), μm
Chocolate clay	3.15	17.1	42.4
Microsilica	0.15	0.46	86.4

**Table 5:** Half-beam manufacturing parameters.

Print Path Length (m)	Layer Height (mm)	Layer Width (mm)	Printing Velocity (mm/s)	Printing Time (min)	Material Volume (L)
753	10	25	95	134	185

**Table 6:** Printing statistics of optimized velocity with accelerator-enhanced mortar mixtures.

Mix Annotation	0% Accelerator	1% Accelerator	1.3% Accelerator
Optimized printing velocity (mm/s)	95	165	195
Printing time (min)	134	77	65
Efficiency factor	1	1.7	2.1

**Table 7:** Printing statistics of optimized velocity with fiber-enhanced mortar mixtures.

Mix Annotation	0% Fibers	0.3% Fibers	0.375% Fibers	0.45% Fibers
Optimized printing velocity (mm/s)	95	140	155	230
Printing time (min)	134	91	82	55
Efficiency factor	1	1.5	1.6	2.4

## Data Availability

The original contributions presented in the study are included in the article, further inquiries can be directed to the corresponding author/s.
